# Plasma Clusterin Concentrations May Predict Resistance to Intravenous Immunoglobulin in Patients with Kawasaki Disease

**DOI:** 10.1155/2013/382523

**Published:** 2013-07-15

**Authors:** Mei-Chen Ou-Yang, Ho-Chang Kuo, I-Chun Lin, Jiunn-Ming Sheen, Fu-Chen Huang, Chih-Cheng Chen, Ying-Hsien Huang, Ying-Jui Lin, Hong-Ren Yu

**Affiliations:** Department of Pediatrics, Kaohsiung Chang Gung Memorial Hospital, College of Medicine, Chang Gung University, 123 Ta-Pei Road, Niao-Sung, Kaohsiung 833, Taiwan

## Abstract

Kawasaki disease (KD) is an acute febrile vasculitic syndrome of early childhood often complicated by coronary artery lesion that drastically reduces the quality of life. The study aimed to identify a reliable marker for predicting nonresponsiveness to the first course of intravenous immunoglobulin (IVIG) in KD patients. A total of 63 patients with KD were enrolled in the study (IVIG response, 58; IVIG resistance, 5). Plasma samples were collected before and after IVIG infusion for measurement of biomarkers. Patients' clinical characteristics and laboratory data were also analyzed. A receiver operating characteristic curve was generated to identify a cut-off value for predicting IVIG resistance. Among the biomarkers, the difference in plasma clusterin concentrations before and after IVIG infusion (CLUSTER 12) was significantly related to IVIG resistance (*P* = 0.040; 95% confidence interval (CI): −25.8% to −6.0%). Using a CLUSTER 12 cut-off value of <8.52 mg/L, the odds ratio for IVIG resistance was 11.467 (95% CI: 1.186 to 110.853). Patients with plasma CLUSTER 12 concentrations >8.52 mg/L had a much higher risk of IVIG resistance than those with CLUSTER 12 concentrations <8.52 mg/L. Plasma clusterin concentration shows promise as a candidate biomarker for predicting IVIG resistance in patients with KD.

## 1. Introduction

Kawasaki disease (KD) is an acute febrile vasculitic syndrome that occurs in early childhood. KD is the leading cause of acquired heart disease and is often associated with coronary artery involvement. Of note, KD complicated by coronary artery lesion (CAL) can have a significant negative impact on quality of life in surviving patients. Intravenous immunoglobulin (IVIG) infusion is the gold standard treatment for KD; however, approximately 10% to 20% of patients do not become afebrile or develop recurrent fever after the first course of IVIG [1–4]. Unresponsiveness to IVIG is also thought to increase the risk of CAL [5]. Thus, identifying factors that cause IVIG resistance may help reduce the occurrence of CAL. 

Numerous studies have reported on risk factors for the development of CAL [6–13]. To date, however, no consistent and reliable criterion has been determined for identifying children most at risk for IVIG resistance and the development of CAL. In a previous study [14, 15], a unique proteomic profile, including increased or decreased fibrinogen, alpha-1-antitrypsin (A1AT), clusterin, and immunoglobulin free light chains, was found to be associated with KD. Among these biomarkers, plasma clusterin is a potential biomarker of KD for predicting the occurrence of CAL. The aim of this study was to identify a reliable biomarker for predicting nonresponsiveness to an initial course of IVIG in patients with KD.

## 2. Material and Methods

### 2.1. Patients

Patients with KD who were admitted to Chang Gung Children's Hospital (Kaohsiung, Taiwan) from February 2008 to March 2011 and treated with IVIG (2 g/kg), followed by low-dose aspirin (3–5 mg/kg/day as a single daily dose) until all signs of inflammation had resolved, were enrolled in the study.

The patients' clinical characteristics and laboratory data were recorded. Plasma samples were collected within 24 h before and after IVIG treatment. These samples were aliquoted and stored at −80°C until further evaluation.

Patients received regular heart echocardiography examinations during the acute febrile stage and thereafter every two weeks for three months. CAL was defined by an internal coronary artery diameter of ≤3 mm (4 mm if the subject was >5 years old) or a segment internal diameter that was at least 1.5 times greater than that of an adjacent segment [16]. IVIG resistance was defined as the return of fever associated with one or more of the original symptoms (that led to the diagnosis of KD) within 48 to 72 hours after initial IVIG treatment [17].

The Institutional Review Board of Chang Gung Memorial Hospital approved the study protocol, which was performed in accordance with the 1964 Declaration of Helsinki. All patients' parents provided written informed consent.

### 2.2. Biomarker Enzyme-Linked Immunosorbent Assays

The plasma concentrations of A1AT, clusterin, fibrinogen, and human immunoglobulin free light chains kappa and lambda were measured by enzyme-linked immunosorbent assay in accordance with the manufacturers' instructions: A1AT (GenWay Biotech, San Diego, CA), clusterin (secreted form) (AdipoGen, Seoul, Republic of Korea), fibrinogen (AssayPro, Charles, MO), and human immunoglobulin free light chains kappa and lambda (BioVendor Laboratory Medicine, Modrice, Czech Republic). We chose to measure the concentrations of these proteins based on previous proteomic profiling.

### 2.3. Statistical Analysis

To evaluate the relationship between biomarkers and IVIG resistance, the patients were divided into two groups: IVIG responders and IVIG nonresponders. Continuous variables are expressed as mean ± standard deviation. Statistical comparisons between the two groups were made using the Wilcoxon rank sum test or Kruskal Wallis test, where appropriate. A receiver operating characteristic (ROC) curve was generated to identify a clusterin cut-off value for predicting IVIG resistance. The specificity and sensitivity of the ROC were calculated with 95% confidence intervals (CIs). The optimal cut-off value was defined as the highest Youden index ((specificity + sensitivity) − 1).

The associations between the biomarkers and IVIG resistance were determined by multivariate logistic regression. Forward selection was used to obtain a final model. Odds ratios were adjusted for clinical characteristics and laboratory data were obtained for each potential biologic marker using IVIG resistance as the dependent variable and the biomarker (the difference in the plasma clusterin concentration between before and after IVIG infusion (CLUSTER 12) was categorized as either ≤8.52 or >8.52 mg/L) as the independent variable. A *P* value <0.05 was considered to be statistically significant. All statistical analyses were performed using SPSS for Windows XP (SPSS, Chicago, IL).

## 3. Results

During the study period, 63 patients with KD were enrolled and treated with IVIG. Among these patients, 58 had a response to initial IVIG therapy and 5 did not. The demographic and clinical characteristics of the 63 patients included in the study are summarized in Table 1.

There were no differences between the IVIG response and resistance groups in terms of age, sex, hemoglobin, total leukocyte count, segment, lymphocyte, monocyte, eosinophil, basophil, platelet count, aspartate aminotransferase, alanine aminotransferase, and C-reactive protein. There was a significant difference in CAL (Table 2).

Table 3 summarizes the plasma biomarkers for each group of patients before and after IVIG. Some factors were further analyzed to determine if they were related to IVIG resistance. Among these factors, the difference in the plasma clusterin concentration before and after IVIG infusion (CLUSTER 12) was significantly related to IVIG resistance (*P* = 0.040; 95% CI: −25.8% to −6.0%). Therefore, patients with lower CLUSTER 12 values had less IVIG resistance than patients with higher CLUSTER 12 values.

**Figure 1:**
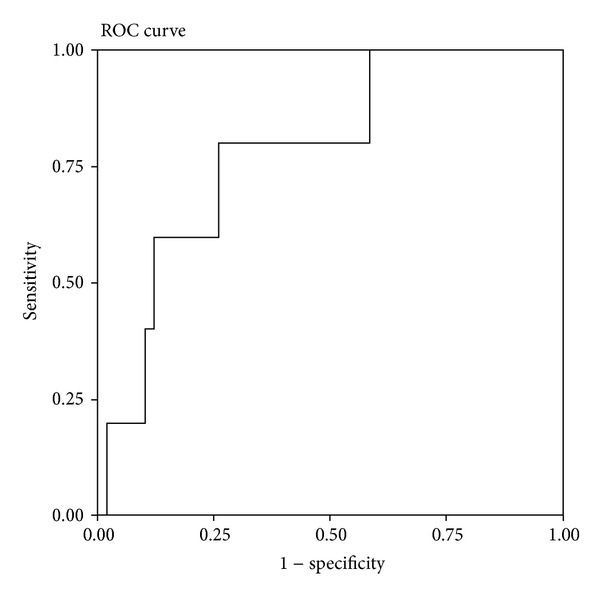
Receiver operating characteristic curve of different plasma clusterin concentrations before and after intravenous immunoglobulin (IVIG) infusion (CLUSTER 12) for the prediction of IVIG resistance in patients (*N* = 68) with Kawasaki disease.

**Figure 2:**
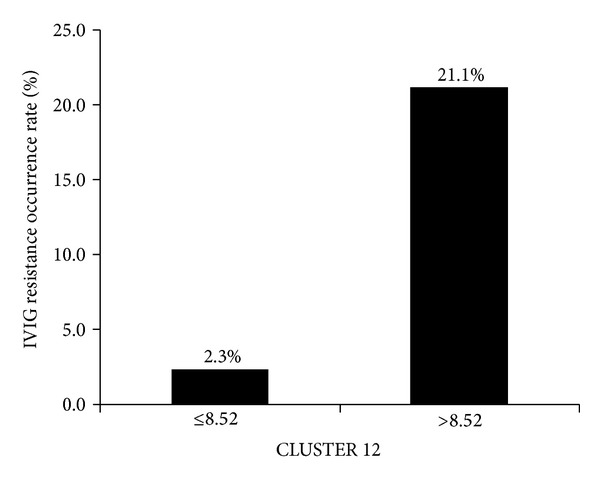
Rate of intravenous immunoglobulin resistance in patients with Kawasaki disease by plasma CLUSTER 12 concentration (≤8.52 or >8.52 mg/L).

To identify the optimal plasma CLUSTER 12 concentration for predicting IVIG resistance in patients with KD, a cut-off value was determined. Using ROC curve analysis, an area under the ROC curve of 0.78 was determined (Figure 1). The optimal cut-off value for predicting IVIG resistance according to the maximum Youden Index was 8.52 mg/L (80.0% sensitivity and 74.1% specificity). The rate of IVIG resistance in patients with a plasma CLUSTER 12 concentration ≤8.52 mg/L was lower than in patients with a plasma CLUSTER 12 concentration >8.52 mg/L (2.30% versus 21.1%, Figure 2).

Using a CLUSTER 12 cut-off value of ≤8.52 mg/L, the odds ratio for IVIG resistance was 11.467 (95% CI: 1.186 to 110.853). Thus patients with a plasma CLUSTER 12 concentration >8.52 mg/L had 11.47-fold higher risk of IVIG resistance than patients with a CLUSTER 12 concentration ≤8.52 mg/L.

## 4. Discussion

KD complicated by CAL can significantly impair the quality of life in surviving patients. Preventing serious cardiac complications is the primary goal of treatment in KD. Unfortunately, some patients experience IVIG resistance, which is associated with poor coronary artery outcomes. Developing an effective strategy for the early detection of resistance to IVIG treatment in patients with KD is crucial. Although several predictive biomarkers have been described [18, 19], there have been no consistent and reliable criteria reported for identifying children most at risk of IVIG resistance and developing CAL. In the present study, we assessed several important biomarkers in an effort to identify predictors of a nonresponse to initial IVIG. Our results show that the difference between plasma clusterin concentrations before and after IVIG infusion (CLUSTER 12) was significantly related to IVIG resistance. Using a cut-off value of CLUSTER 12 ≤8.52 mg/L, only 2.30% of patients with KD were IVIG resistant in our cohort. Thus, a CLUSTER 12 concentration >8.52 mg/L may be a good predictor of IVIG resistance in patients with KD.

In previous reports [1, 20, 21], patients who were IVIG resistant were found to have a higher risk of coronary artery abnormalities. In the present study, 92% (58/63) of patients responded to initial IVIG therapy and 8% (5/63) of patients did not. Of note, we found that the presence of CAL was significantly related to IVIG resistance (*P* < 0.001). Patients who were IVIG resistant had a higher probability (5/5, 100%) of developing coronary artery abnormalities. Therefore, identifying IVIG resistance in patients with KD patients is critical so that these patients can be prescribed more aggressive initial therapy, such as administration of pulsed intravenous methylprednisolone (along with initial IVIG), to help prevent coronary artery complications [22].

Clusterin (also named apolipoprotein J) has been associated with inflammation and lipid metabolism, as well as the pathophysiologic sequelae of these conditions, such as cardiovascular disease and malignancy. Recent studies have found that clusterin may play a role in vascular smooth muscle migration and proliferation and exert a cardioprotective effect on cardiomyocytes and coronary vessels [23, 24]. Previous studies have established that plasma clusterin concentrations are higher in patients with KD than in other febrile patients [15] and that low clusterin concentrations are restricted to patients with KD who have CAL. An early biomarker (clusterin < 12 mg/L) before IVIG treatment has been proposed to predict the occurrence of CAL. The present study shows that IVIG resistance is higher in patients with KD who have plasma CLUSTER 12 concentrations >8.52 mg/L. This implies that patients with KD who have plasma CLUSTER 12 concentrations >8.52 mg/L may need more intensive treatment to prevent the need for retreatment and the occurrence of coronary artery complications.

Although altered plasma clusterin concentrations have been reported in patients with coronary heart disease, the pathophysiological mechanism underlying these alterations and the association with KD remains unknown [25–27]. Results from our study show that the difference between plasma clusterin concentrations before and after IVIG infusion may be a candidate for predicting IVIG resistance. We found that plasma CLUSTER 12 concentrations >8.52 mg/L were associated with a much higher risk of IVIG resistance in patients with KD. Our finding that plasma clusterin concentrations are elevated after IVIG infusion in patients who are IVIG resistant suggests that clusterin may also play a role as an inflammatory mediator with a cytoprotective effect. van Dijk et al. [23] previously reported that intravenous clusterin administration reduced myocardial infarct size in rats. In addition, previous studies have also shown that clusterin mRNA and protein concentrations are upregulated in models of myocarditis and ischemia and localized in necrotic areas of cardiomyocytes after myocardial infarction. Further, clusterin has been demonstrated to protect cardiomyocytes from ischemia-induced cell death in vitro [28, 29].

In summary, we suggest that clusterin may serve as a potential novel therapeutic target in the treatment of KD. Further large-scale studies are required to validate this predictive marker and determine the pathophysiologic role of clusterin in KD.

## Figures and Tables

**Table 1:** Demographic and clinical characteristics of patients with Kawasaki disease.

Characteristic	*N* = 63
Age (months)	19.86 ± 18.72
Sex (M : F)	40 : 23
Days of fever before admission	4.28 ± 3.12
IVIG resistance rate	5/58
Hemoglobin	10.88 ± 1.32
WBC	12583.3 ± 3132.6
Segment (%)	63.70 ± 10.38
Lymphocyte (%)	22.52 ± 9.46
Monocyte (%)	6.03 ± 3.43
Eosinophil (%)	3.15 ± 2.87
Basophil (%)	0.25 ± 1.07
AST (U/L)	87.53 ± 63.72
ALT (U/L)	64.25 ± 60.59
Platelet (×10^4^/mm^3^)	35.75 ± 11.68
CRP (mg/L)	101.53 ± 78.23

Data are presented as mean standard deviation for quantitative variables.

M : F: Male : Female; WBC: white blood cells; AST: aspartate aminotransferase; ALT: alanine aminotransferase; CRP: C-reactive protein.

**Table 2:** Demographic and clinical characteristics of patients with Kawasaki disease who had a response to IVIG or were IVIG resistant.

Variable	IVIG response (*n* = 58)	IVIG resistance (*n* = 5)	*P* value
Age (months)	21.66 ± 19.12	12.28 ± 6.72	0.491
Sex (M : F)	37 : 21	2 : 3	0.866
Days of fever before admission	5.02 ± 1.82	4.89 ± 2.32	0.453
CAL	7/58	5/5	<0.001*
Hemoglobin	10.54 ± 1.38	11.21 ± 2.11	0.432
WBC	12262.9 ± 3163.4	13100.9 ± 3528.3	0.520
Segment (%)	63.22 ± 10.86	64.21 ± 11.10	0.273
Lymphocyte (%)	23.04 ± 8.23	22.31 ± 9.15	0.172
Monocyte (%)	5.93 ± 3.12	6.14 ± 3.82	0.383
Eosinophil (%)	3.32 ± 3.15	2.62 ± 2.76	0.482
Basophil (%)	0.22 ± 0.42	0.32 ± 1.21	0.712
AST (U/L)	80.56 ± 51.38	95.32 ± 69.22	0.354
Platelet (×10^4^/mm^3^)	35.36 ± 11.41	35.84 ± 14.71	0.235
CRP(mg/L)	95.12 ± 53.29	137.34 ± 136.38	0.389
Kappa (mg/L)	4.74 ± 5.19	5.21 ± 1.49	0.844
Lambda (mg/L)	12.96 ± 12.01	15.02 ± 8.99	0.714
A1AT (mg/dL)	186.51 ± 29.34	198.61 ± 18.49	0.375

Data are presented as mean ± standard deviation for quantitative variables. CAL: coronary artery lesion; WBC: white blood cells; AST: aspartate aminotransferase; ALT: alanine aminotransferase; CRP: C-reactive protein. A1AT: alpha-1-antitrypsin. *P < 0.05 was considered statistically significant difference.

**Table 3:** Plasma biomarkers in patients with Kawasaki disease who had a response to IVIG or were IVIG resistant.

Biomarker	Response (*n* = 58)	Resistance (*n* = 5)	*P* value
CLUSTER 1	25.02 ± 12.33	16.44 ± 6.59	0.132
CLUSTER 2	23.09 ± 12.59	27.70 ± 10.14	0.429
CLUSTER 12	−1.93 ± 13.66	11.26 ± 11.17	0.040*
CLUSTER 012	1.33 ± 0.90	0.64 ± 0.35	0.097
FIB 1	1141.68 ± 795.77	803.94 ± 185.89	0.351
FIB 2	1513.52 ± 1122.97	1258.25 ± 946.08	0.624
FIB 12	371.85 ± 726.86	454.31 ± 895.55	0.812
FIB 012	0.83 ± 0.34	0.82 ± 0.31	0.934

CLUSTER 1 (mg/L): plasma clusterin concentration before IVIG treatment; CLUSTER 2 (mg/L): plasma clusterin concentration after IVIG treatment; CLUSTER 12 (mg/L): CLUSTER 2 − CLUSTER 1; CLUSTER 012: CLUSTER 2/CLUSTER 1; FIB 1 (mg/dL): plasma fibrinogen concentration before IVIG treatment; FIB 2 (mg/dL): plasma fibrinogen concentration after IVIG treatment; FIB 12 (mg/dL): FIB 2 – FIB 1; FIB 012: FIB 2/FIB 1. *P < 0.05 was considered statistically significant difference.

## References

[1] Burns JC, Capparelli EV, Brown JA, Newburger JW, Glode MP (1998). Intravenous gamma-globulin treatment and retreatment in Kawasaki disease. US/Canadian Kawasaki Syndrome Study Group. *Pediatric Infectious Disease Journal*.

[2] Burns JC, Glodé MP (2004). Kawasaki syndrome. *The Lancet*.

[3] Burns JC (2007). The riddle of Kawasaki disease. *New England Journal of Medicine*.

[4] Wallace CA, French JW, Kahn SJ, Sherry DD (2000). Initial intravenous gammaglobulin treatment failure in Kawasaki disease. *Pediatrics*.

[5] Durongpisitkul K, Soongswang J, Laohaprasitiporn D, Nana A, Prachuabmoh C, Kangkagate C (2003). Immunoglobulin failure and retreatment in Kawasaki disease. *Pediatric Cardiology*.

[6] Ban JY, Yoon KL, Kim SK, Kang S, Chung J-H (2009). Promoter polymorphism (rs3755724, −55C/T) of tissue inhibitor of metalloproteinase 4 (TIMP4) as a risk factor for Kawasaki disease with coronary artery lesions in a Korean population. *Pediatric Cardiology*.

[7] Biezeveld MH, Kuipers IM, Geissler J (2003). Association of mannose-binding lectin genotype with cardiovascular abnormalities in Kawasaki disease. *The Lancet*.

[8] Kim T, Choi W, Woo C-W (2007). Predictive risk factors for coronary artery abnormalities in Kawasaki disease. *European Journal of Pediatrics*.

[9] Nishimura S, Zaitsu M, Hara M (2003). A polymorphism in the promoter of the CD14 gene (CD14/−159) is associated with the development of coronary artery lesions in patients with kawasaki disease. *Journal of Pediatrics*.

[10] Nofech-Mozes Y, Garty B-Z (2003). Thrombocytopenia in Kawasaki disease: a risk factor for the development of coronary artery aneurysms. *Pediatric Hematology and Oncology*.

[11] Ohno T, Igarashi H, Inoue K, Akazawa K, Joho K, Hara T (2000). Serum vascular endothelial growth factor: a new predictive indicator for the occurrence of coronary artery lesions in Kawasaki disease. *European Journal of Pediatrics*.

[12] Salo E, Pesonen E, Viikari J (1991). Serum cholesterol levels during and after Kawasaki disease. *Journal of Pediatrics*.

[13] Song D, Yeo Y, Ha K (2009). Risk factors for Kawasaki disease-associated coronary abnormalities differ depending on age. *European Journal of Pediatrics*.

[14] Yu H-R, Kuo H-C, Huang E-Y (2010). Plasma clusterin levels in predicting the occurrence of coronary artery lesions in patients with kawasaki disease. *Pediatric Cardiology*.

[15] Yu H-R, Kuo H-C, Sheen J-M (2009). A unique plasma proteomic profiling with imbalanced fibrinogen cascade in patients with Kawasaki disease. *Pediatric Allergy and Immunology*.

[16] Kuo HC, Wang CL, Liang CD (2009). Association of lower eosinophil-related T helper 2 (Th2) cytokines with coronary artery lesions in Kawasaki disease. *Pediatric Allergy and Immunology*.

[17] Kuo H-C, Yang KD, Liang C-D (2007). The relationship of eosinophilia to intravenous immunoglobulin treatment failure in Kawasaki disease. *Pediatric Allergy and Immunology*.

[18] Fury W, Tremoulet AH, Watson VE (2010). Transcript abundance patterns in Kawasaki disease patients with intravenous immunoglobulin resistance. *Human Immunology*.

[19] Suzuki H, Suenaga T, Takeuchi T, Shibuta S, Yoshikawa N (2010). Marker of T-cell activation is elevated in refractory Kawasaki disease. *Pediatrics International*.

[20] Tremoulet AH, Best BM, Song S (2008). Resistance to intravenous immunoglobulin in children with Kawasaki disease. *Journal of Pediatrics*.

[21] Rigante D, Valentini P, Rizzo D (2010). Responsiveness to intravenous immunoglobulins and occurrence of coronary artery abnormalities in a single-center cohort of Italian patients with Kawasaki syndrome. *Rheumatology International*.

[22] Newburger JW, Sleeper LA, McCrindle BW (2007). Randomized trial of pulsed corticosteroid therapy for primary treatment of Kawasaki Disease. *New England Journal of Medicine*.

[23] van Dijk A, Vermond RA, Krijnen PAJ (2010). Intravenous clusterin administration reduces myocardial infarct size in rats. *European Journal of Clinical Investigation*.

[24] ter Weeme M, Vonk ABA, Kupreishvili K (2010). Activated complement is more extensively present in diseased aortic valves than naturally occurring complement inhibitors: a sign of ongoing inflammation. *European Journal of Clinical Investigation*.

[25] Poulakou MV, Paraskevas KI, Wilson MR (2008). Apolipoprotein J and leptin levels in patients with coronary heart disease. *In Vivo*.

[26] Trougakos IP, Poulakou M, Stathatos M, Chalikia A, Melidonis A, Gonos ES (2002). Serum levels of the senescence biomarker clusterin/apolipoprotein J increase significantly in diabetes type II and during development of coronary heart disease or at myocardial infarction. *Experimental Gerontology*.

[27] Kujiraoka T, Hattori H, Miwa Y (2006). Serum apolipoprotein J in health, coronary heart disease and type 2 diabetes mellitus. *Journal of Atherosclerosis and Thrombosis*.

[28] Swertfeger DK, Witte DP, Stuart WD, Rockman HA, Harmony JAK (1996). Apolipoprotein J/clusterin induction in myocarditis. A localized response gene to myocardial injury. *American Journal of Pathology*.

[29] Krijnen PAJ, Cillessen SAGM, Manoe R (2005). Clusterin: a protective mediator for ischemic cardiomyocytes?. *American Journal of Physiology—Heart and Circulatory Physiology*.

